# Quercetin-conjugated superparamagnetic iron oxide nanoparticles (QCSPIONs) increases Nrf2 expression via miR-27a mediation to prevent memory dysfunction in diabetic rats

**DOI:** 10.1038/s41598-020-71971-2

**Published:** 2020-09-29

**Authors:** Shiva Ebrahimpour, Seyedeh Bahar Shahidi, Mahnoosh Abbasi, Zahra Tavakoli, Abolghasem Esmaeili

**Affiliations:** Department of Cell and Molecular Biology & Microbiology, Faculty of Biological Science and Technology, University of Isfahan, HezarJarib Street, P.O. Box: 8174673441, 81746-73441 Isfahan, Iran

**Keywords:** Drug discovery, Molecular biology, Neuroscience

## Abstract

Oxidative stress is one of the earliest defects involved in the development of diabetes-induced cognitive impairment. Nrf2 is the master regulator of the cellular antioxidant system can be regulated by some microRNAs. The study aimed to evaluate the effects of quercetin (QC) and quercetin-conjugated superparamagnetic iron oxide nanoparticles (QCSPIONs) on Nrf2-controlled antioxidant genes through the redox-sensitive miR-27a. Expression levels of miR-27a, Nrf2, SOD1, GPX1, and CAT were measured by quantitative real-time PCR. Moreover, the oxidative stress parameters including total antioxidant capacity (TAC) and histological alterations were investigated. The expression level of miR-27a was significantly up-regulated in diabetic rats. While expression levels of Nrf2, SOD1, GPX1, and CAT were significantly down-regulated under diabetic condition. Interestingly, QCSPIONs decreased expression level of miR-27a and subsequently enhanced the expression levels of Nrf2, SOD1, and CAT to the control level. No significant difference was observed in the expression level of GPX1. Besides, QC in pure and especially conjugated form was able to normalize TAC and regenerate pathological lesions in STZ-diabetic rats. Our result demonstrates that QCSPIONs as an effective combined therapy can decrease miR-27a expression, which in turn increases the Nrf2 expression and responsive antioxidant genes, resulting in improvement of memory dysfunction in diabetic rats.

## Introduction

Diabetes mellitus (DM) is the most common chronic metabolic disorder characterized by persistent hyperglycemia associated with lipid, protein, and carbohydrate metabolism abnormalities^1^. Diabetes is also related to long-term complications affecting the kidneys, retina, arteries, cardiovascular system, and nervous system^2^. The chronic and continuous hyperglycemia produced free radicals and reactive oxygen species (ROS) which trigger oxidative stress. Intracellular oxidative stress results in lipid peroxidation, DNA damage, protein degradation, and exhaustion of the antioxidant defense systems^3^. It has been shown the production of AGEs, auto-oxidation of glucose, alteration in antioxidant enzymes, and suppression of immune response result in exacerbated oxidative stress in diabetic patients. Recent studies have shown that oxidative stress caused by diabetes leads to brain vascular complications and cognitive deficits^4^. For example, oxidative stress can lead to Aβ pathology through stimulation of amyloid precursor protein (APP) gene expression or by modulation of its processing via γ-secretase expression^5^. The nuclear factor-erythroid 2-(NF-E2-) related factor 2 (Nrf2) is a redox-sensitive transcription factor that protects cells from oxidative stress-induced cell death and modulates redox signaling pathway through its ability to regulate phase II detoxifying enzymes and antioxidant proteins^6–8^. Nrf2 controls the expression of an array antioxidant response element-dependent genes, including heme oxygenase-1 (HO1), NADPH quinone dehydrogenase 1 (NQO1), glutathione peroxidase 1 (GPX1), superoxide dismutase 1 (SOD1) and catalase (CAT) resulting in the inhibition of oxidative stress and accumulation of lipid in DM^9,10^. Dysregulation of Nrf2 has been involved in diverse pathophysiological processes including cancer, alcoholic liver disease, chronic obstructive pulmonary disease, diabetes, and neurodegenerative diseases^6^. Despite the regulation of Nrf2 via Kelch-like ECH-associated protein 1 (Keap1)-dependent mechanism, recent studies demonstrate that Nrf2 can be regulated by some microRNAs^6,11^. In silico analysis of the miRBase resource by Papp et al. indicated that 85 microRNAs are predicted to bind to Nrf2 mRNA to downregulate its translation^12^. microRNA-27a is one of the redox-sensitive microRNAs or “redoximiRs” which plays a direct role in the downregulation of Nrf2 and modulation of Nrf2-driven antioxidant gene expression. It has been shown that enhancement of miR-27a leads to suppression of Nrf2 and further, repression of Nrf2-controlled antioxidant genes in diabetic embryopathy^13^. In addition, it has been reported that upregulation microRNAs cause neurodegeneration by reducing the levels of Nrf2^14^. Therefore, Nrf2 activators could be therapeutically effective in treating diabetic complications.

Quercetin (QC) (2-(3,4-dihydroxyphenyl)-3,5,7-trihydroxy-4H-chromen-4-one) is one of the most abundant flavonoid compounds naturally found in the human diet such as vegetables, fruits, wines, black and green teas and cereal^15^. This bioflavonoid shows a wide range of biological and health-promoting effects, such as free radical scavenging, anti-inflammatory, antidiabetic, anticancer, and antibacterial activities^16,17^. QC acts as a direct antioxidant that scavenges free radicals and chelates metallic ions due to the presence of two pharmacophores within its structure that have the optimal configuration for free radical scavenging^18,19^. In addition, QC indirectly plays an important role in counteracting oxidative stress by modulating the Nrf2-ARE pathway^20^. Emerging studies reported that QC presented beneficial effects against various diseases not only through influencing directly gene expression at an epigenetic or transcriptional level but also by modulating miRNAs as part of the post-transcriptional regulation of genes^21^. Despite these favorable properties, its bioavailability to the brain is low owing to poor solubility, stability, and distribution so various conjugates of QC have been developed to overcome these limitations^16,22^. In a previous report, we revealed that oral delivery of quercetin-conjugated superparamagnetic iron oxide nanoparticles (QCSPIONs) in the range of 30–50 nm had significantly better efficacy on the improvement of memory performance in diabetic rats compared to pure QC and proposed an effective combined therapy in the maintenance of learning and memory^23^.

The current report focuses on the miR-27a/Nrf2 antioxidant pathway as one of the molecular mechanisms underlying the neuroprotective effect of QCSPIONs against memory dysfunction. Although a number of new studies have considered miR-27a as a molecular target for QC in pathological conditions including colon cancer^24^, renal cancer^25^, and colorectal cancer^26^, to the best of our knowledge no previous study has investigated the effect of QC on microRNA-27a in diabetes-related cognitive impairment. Thus, this study aimed to compare the effect of pure QC and QCSPIONs on the expression of miR-27a, Nrf2, SOD1, GPX1, and CAT genes as well as the histological alteration in multiple low-dose streptozotocin (MLDS)-induced diabetic rats.

## Results

### Characterization of QCSPIONs

The Fourier transform infrared (FTIR) spectrum of QU and dextran-coated SPIONs revealed the presence of the OH stretching vibration of the hydroxyl groups, the Fe–O vibration frequency of magnetite spinel structure in SPIONs and C=O, OH groups, and a region corresponding to C–O stretching in QC as was previously reported^23,27^. The QCSPIONs spectrum showed the existence of the stretching vibrations of hydroxyl groups and other characteristic bands, that approved the effective conjugation of QC on the superparamagnetic nanoparticles. The X-ray diffraction (XRD) confirmed the presence of the crystalline structure of the magnetite. This for pure QC showed several different peaks characteristic of high crystalline nature, however, no diversion peaks were detected in QCSPIONs in comparison to pure QC. The external morphology of the QCSPIONs was imagined by scanning electron microscope (SEM) data confirmed a spherical shape of QCSPIONs. Pictures related to this part of the study presented in our former studies^23,27^.

### The content of NPs in the brain

**Figure 1:**
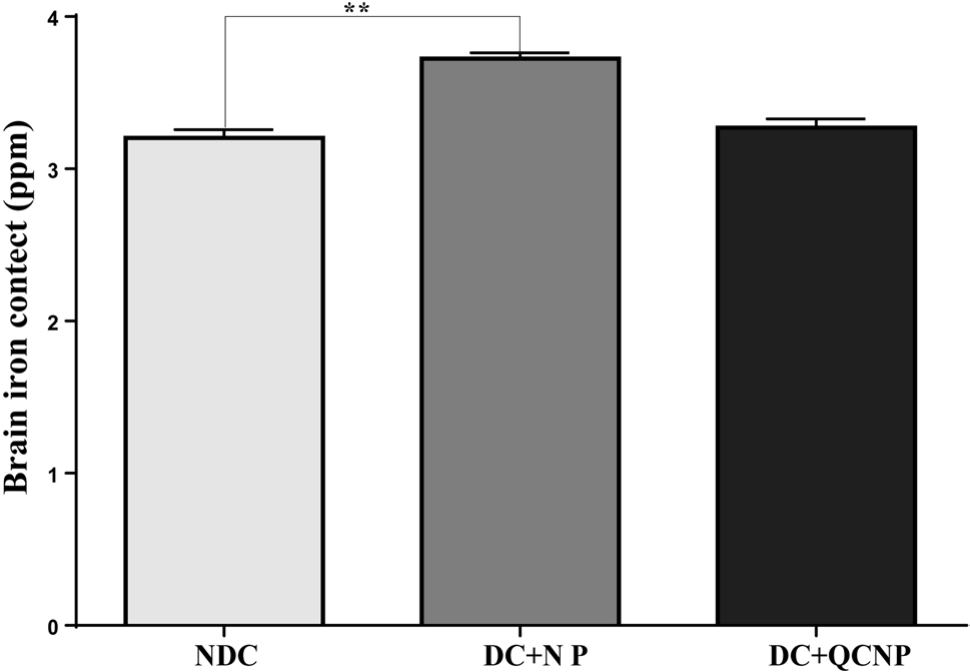
Total iron concentration after oral administration of Fe_3_O_4_ NPs (25 mg/kg) during 35 days in the brain of rats. All values are reported mean ± S.E.M of 3 rats per group. *NDC* non-diabetic control, *DC + NP* diabetic treated with nanoparticle. **P < 0.01 versus diabetic control group. Results were analyzed by using the GraphPad Prism software version 6.07 (https://en.freedownloadmanager.org/users-choice/Graphpad_Prism_6.07_Download.html).

To further verify the uptake of nanoparticles into the brain of rats, the iron accumulations in the hippocampus 35 days after oral administration of SPIONs were quantified by ICP-AES. As shown in Fig. 1, iron content in control rats was 3.203 ± 0.05 ppm whereas in diabetic rats treated with SPIONs was 3.723 ± 0.03 ppm. This data revealed a significant difference in iron contents in control and SPIONs treated rats (P = 0.0015) after 5 days from the last daily gavage. Although the concentration of iron in the hippocampus of the QCSPIONs group was greater than the control, the alteration was not significant which may be due to a lower concentration of iron in the conjugated form.

### Expression levels of hippocampal microRNA and mRNAs

**Figure 2:**
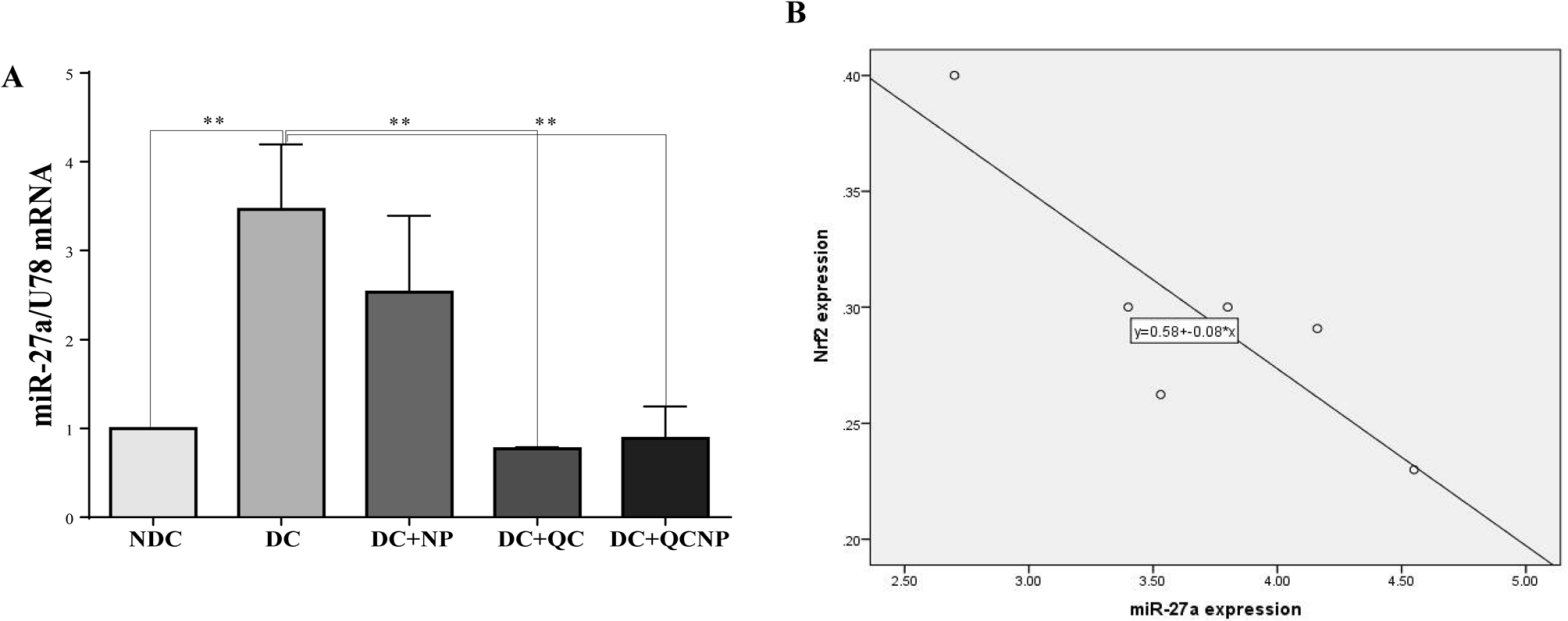
The graph of real-time PCR analysis of (**A**) miR-27a expression in the rat hippocampus, (**B**) Correlation between the expression levels of miR-27a and Nrf2 in DC. Results are expressed as mean ± SEM, n = 4–5 rat per group (**p < 0.01 vs. diabetic group). *QC* quercetin, *NP* nanoparticle, *NDC* non-diabetic control, *DC* diabetic control, *DC* + *NP* diabetic treated with nanoparticle, *DC* + *QC* diabetic treated with quercetin, *DC* + *QCNP* diabetic treated with quercetin nanoparticle. Results were analyzed by using the GraphPad Prism software version 6.07 (https://en.freedownloadmanager.org/users-choice/Graphpad_Prism_6.07_Download.html).

**Figure 3:**
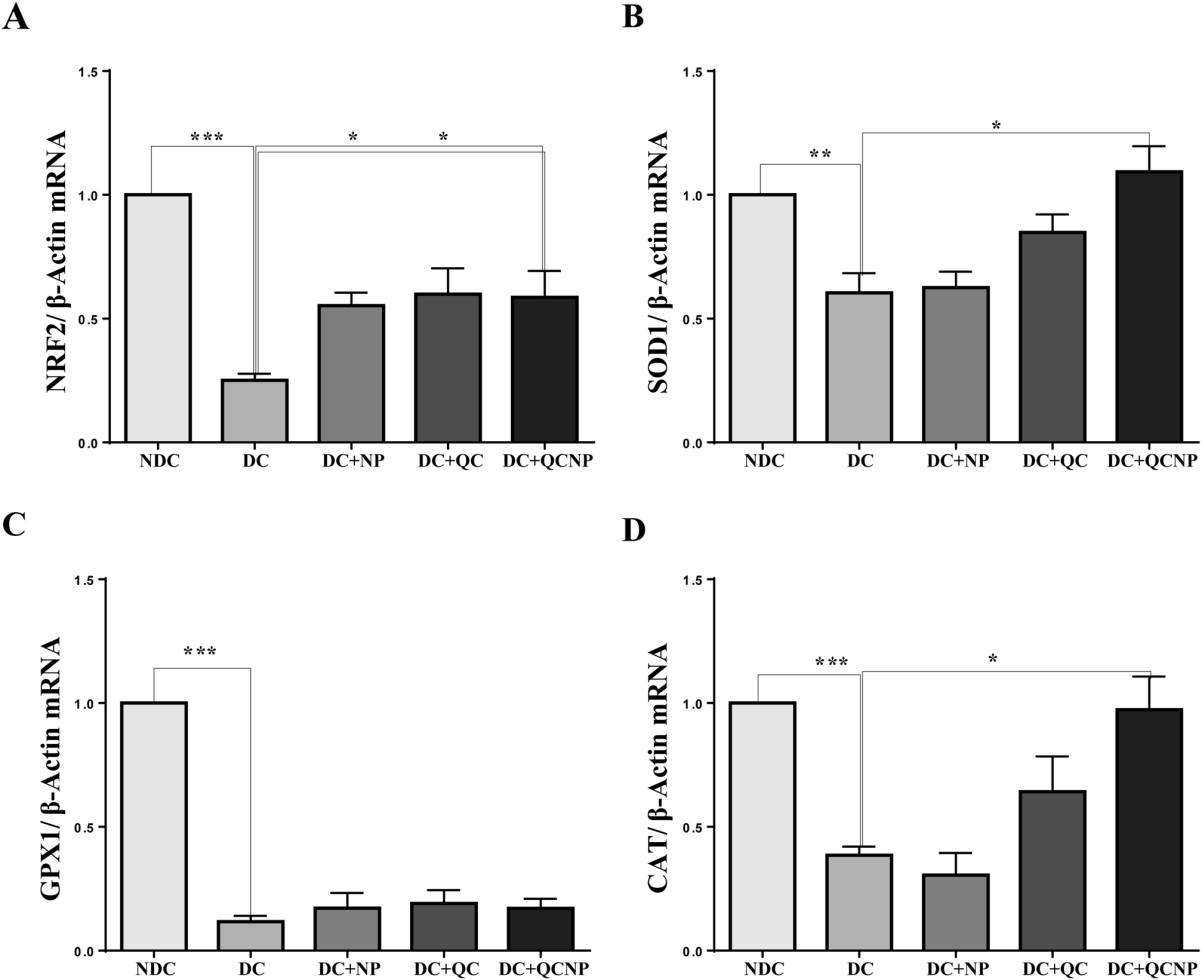
The graph of real-time PCR analysis of mRNAs expression levels of (**A**) Nrf2, (**B**) SOD1, (**C**) GPX1, (**D**) CAT. Results are expressed as mean ± SEM, n = 4–5 rat per group (*p < 0.05, **p < 0.01 and ***p < 0.001 vs. diabetic group). *QC* quercetin, *NP* nanoparticle, *NDC* non-diabetic control, *DC* diabetic control, *DC* + *NP* diabetic treated with nanoparticle, *DC* + *QC* diabetic treated with quercetin, *DC* + *QCNP* diabetic treated with quercetin nanoparticle. Results were analyzed by using the GraphPad Prism software version 6.07 (https://en.freedownloadmanager.org/users-choice/Graphpad_Prism_6.07_Download.html).

The melting curve for miR-27a, miR-U87, Nrf2, SOD1, GPX1, CAT, and B-actin presented a single peak at a temperature range of 80–95, revealing a unique PCR product in each reaction. Two months after the start of the study one-way ANOVA analysis indicated a significant difference in the expression of hippocampal miR-27a level in all of the experimental groups. As shown in Fig. 2, the expression level of miR-27a was up-regulated more than threefold in the STZ group (fold change = 3.46, p = 0.0043), while treatment with QC in pure and conjugated forms markedly decreased miR-27a expression in the hippocampus of diabetic rats (fold change = 0.77, p = 0.0031 for QC and fold change = 0.88, p = 0.0054 for QCNP). As shown in Fig. 3 the levels of Nrf2 and its target genes SOD1, GPX1, and CAT were down-regulated in diabetic rats, however, this down-regulation was reversed by QC treatment. As shown in Fig. 3A, the mRNA level of Nrf2 in the diabetic group was dramatically decreased compared with the control group (fold change = 0.23, p = 0.0001). Regression analysis indicates that there is a significant negative correlation between the expression of miR-27a and Nrf2 (p = 0.029) (Fig. 2B). Post hoc comparison showed an increased effect on the expression of the hippocampal Nrf2 mRNA level in rats who received QC and QCSPIONs during the experiment (fold change = 0.63, p = 0.0511 for QC and fold change = 0.58, p = 0.0385 for QCNP). The SOD1 and CAT mRNA levels were also decreased in STZ rats (fold change = 0.60, p = 0.0077 for SOD1 and fold change = 0.45, p = 0.0001 for CAT), while a significant increase in the expression of these genes was observed in QC (fold change = 0.90, p = 0.0863 for SOD1 and fold change = 0.64, p = 0.1549 for CAT) and QCSPIONs groups (fold change = 1.09, p = 0.0204 for SOD1 and fold change = 0.97, p = 0.0132 for CAT) (Fig. 3B,D). What is interesting in this data is that antioxidant genes mRNA levels in QCSPIONs rats were significantly higher than those that received pure QC. Unexpectedly, the level of expression of GPX1 in the diabetic group showed a very severe decrease (fold change = 0.09, p = 0.0001) so that treatments had no significant effect on its mRNA expression (P = 0.2985) (Fig. 3C).

### Total antioxidant capacity measurement

**Figure 4:**
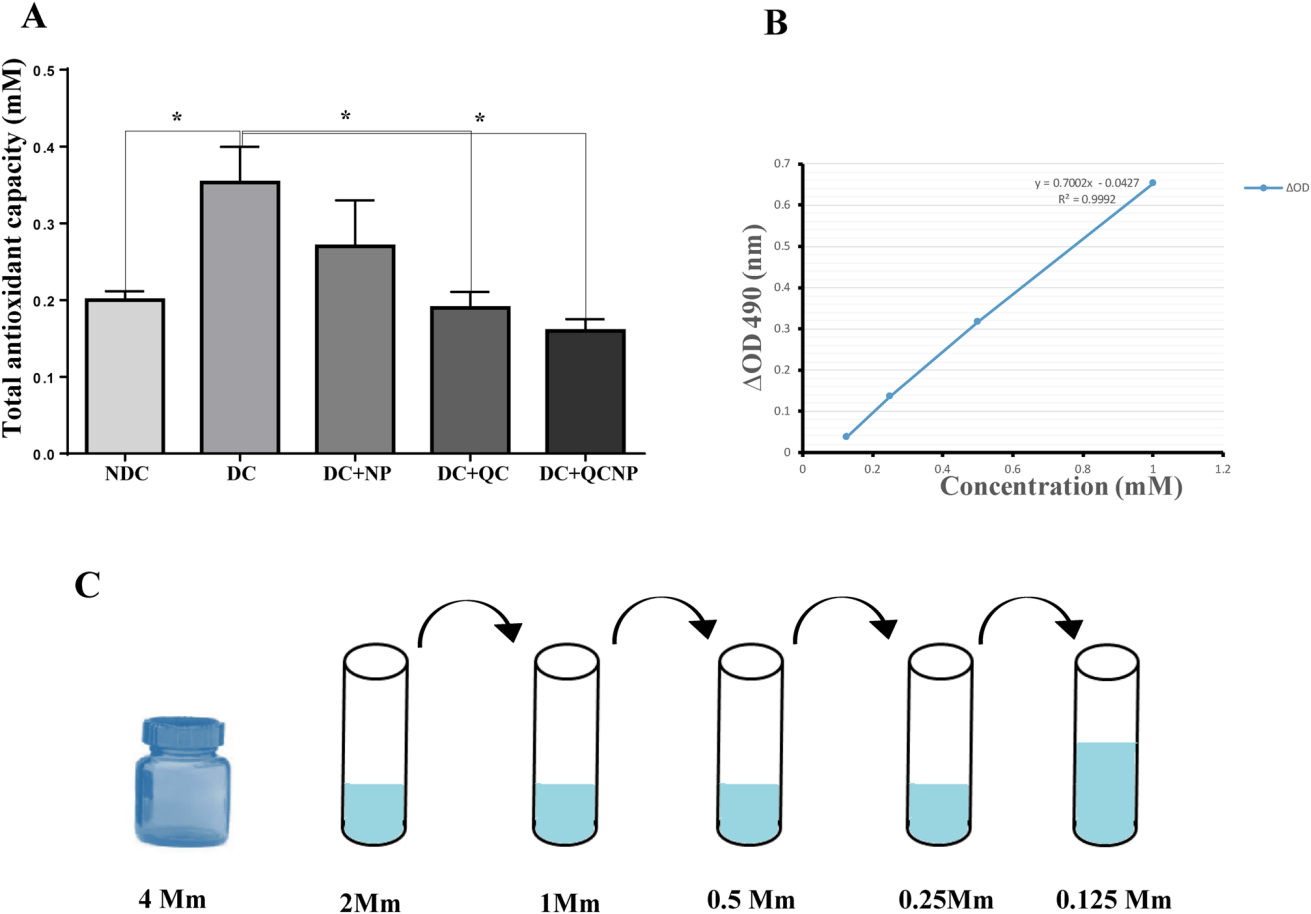
Total antioxidant capacity in the hippocampus of male Wistar rats. (**A**) Comparison of hippocampal total antioxidant capacity (TAC) in non-diabetic control (NDC), diabetic control (DC), diabetic treated with nanoparticle (*DC* + *NP*), diabetic treated with quercetin (*DC* + *QC*), diabetic treated with quercetin nanoparticle (*DC* + *QCNP*) (25 mg/kg for 35 days). (**B**) A typical standard curve of ZellBio GmbH TAC assay kit. (**C**) Schematic picture of the preparation of solutions. Results are expressed as mean ± SEM, n = 4–5 rat per group (*p < 0.05 vs. diabetic group). Results were analyzed by using the GraphPad Prism software version 6.07 (https://en.freedownloadmanager.org/users-choice/Graphpad_Prism_6.07_Download.html).

Figure 4 presents the results obtained from the TAC assay in all experimental groups. Average TAC level in control and diabetic groups was 0.20 ± 0.01 and 0.35 ± 0.04 whereas in diabetic rats treated with QC and QCSPIONs was 0.19 ± 0.02 and 0.16 ± 0.01 respectively. According to the data, there was a significant increase in the total antioxidant level in the diabetic group compared to the control group (p = 0.0332). A significant decreased in the hippocampus concentration of TAC was observed in the QC (p = 0.0330) and QCSPIONs (p = 0.0170) groups compared to the diabetic group. However, the conjugated form of QC revealed relatively more effect on the modulation of the antioxidant capacity of diabetic rats.

### Pathological lesions

**Figure 5:**
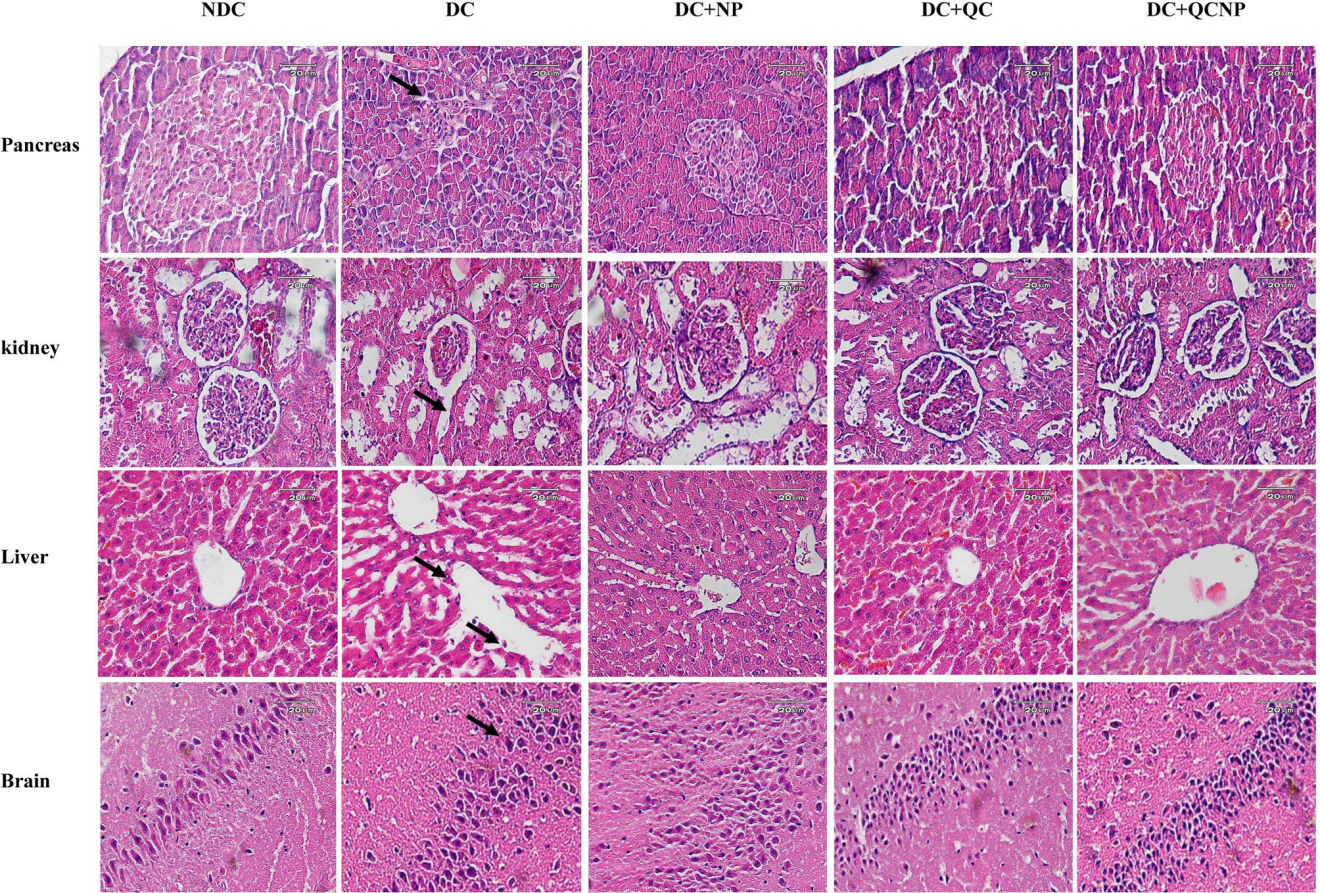
Photomicrographs of pancreas, kidney, liver, and brain sections after treatment with NP, QC, QCNP (H&E staining, magnification 40 ×, scale bar: 20 μm). *H&E* hematoxylin & eosin, N*DC* non-diabetic control, *DC* diabetic control, *DC* + *NP* diabetic treated with nanoparticle, *DC* + *QC* diabetic treated with quercetin, *DC* + *QCNP* diabetic treated with quercetin nanoparticle. Histological evaluation was performed by ImageJ software version 1.52v (https://imagej.en.softonic.com/download).

**Figure 6:**
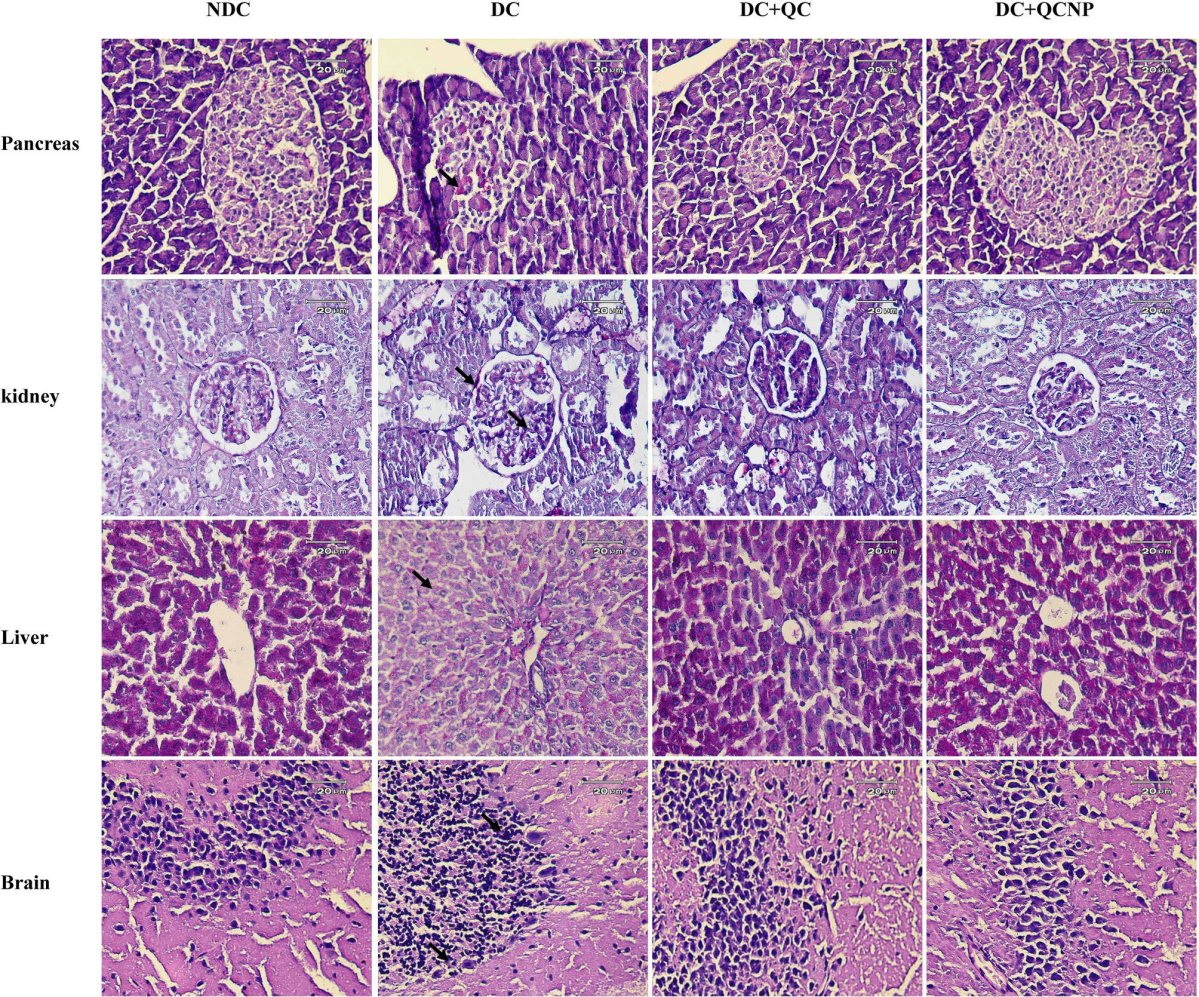
Photomicrographs of pancreas, kidney, liver, and brain sections after treatment with NP, QC, QCNP (PAS staining, magnification 40 ×, scale bar: 20 μm). *PAS* Periodic acid-Schiff, *NDC* non-diabetic control, *DC* diabetic control, *DC* + *NP* diabetic treated with nanoparticle, *DC* + *QC* diabetic treated with quercetin, *DC* + *QCNP* diabetic treated with quercetin nanoparticle. Histological evaluation was performed by ImageJ software version 1.52v (https://imagej.en.softonic.com/download).

Histopathological changes were assessed using H&E and PAS staining (Figs. 5, 6). H&E staining demonstrated small and deformed islets, necrotic cells with pyknotic nuclei and dense eosinophilic cytoplasm. Also, PAS-positive inclusions observed in Langerhans islet cells of the diabetic rats. The pancreas sections from diabetic animals treated with QC and QCSPIONs showed overall remarkable recovery changes toward normal histology. H&E staining showed diabetes-induced renal morphological alterations, including glomeruli deformation, Bowman’s capsule damage, glomeruli tip lesions, and abnormal glomerular capsular space. PAS staining revealed glomerular hypertrophy, the proliferation of mesangial cells, expansive mesangial expansion, and thickening of glomerular basement membrane in diabetic kidney sections. Importantly, these histological alterations induced by streptozotocin (STZ) completely reverted by QC and QCSPIONs treatments. H&E staining of rat liver illustrating the loss of the normal architecture including swelled central veins in different areas with lots of leucocytic infiltrations into the vein and in between vacuolations, irregular lobular pattern and distorted sinusoidal spaces. PAS staining evaluation illustrated a large amount of glycogen storage in normal hepatocytes. On the contrary, intracellular glycogen storage was greatly reduced in the STZ group. Upon QC and QCSPIONs treatments a regeneration in hepatic lobular and an escalation in glycogen reserve was observed. H&E staining revealed irregular neurons in dentate gyri of diabetic rats and pyknotic nuclei in many granular cells, suggesting apoptosis. In addition, PAS staining revealed lipofuscin accumulation in the hippocampus region. In QC and QCSPIONs treated groups, most of the granular neurons were considered normal.

## Discussion

There is growing evidence that diabetes inclines to cognitive decline leading to dementia in both animal models and humans with both type 1 and type 2 diabetes^28^. Diabetes-related cognitive dysfunction can seriously challenge the demand for future health resources, so understanding the pathophysiology of the disorder and identifying molecular pathways that can lead to improved therapy in the future are urgent^28^. Recent studies provide evidence that naturally derived phenolic substances, because of their biological properties, may be unique nutraceuticals and represent supplementary treatments for various aspects of DM^29^. In a previous study, we use QC as a phyto-derived bioactive compound and QC conjugated with SPIONs as a nano-based delivery system to prevent cognitive dysfunction in diabetic rats. We showed that diabetes causes memory impairment during 45 days in male Wistar rats. Memory performance parameters like spatial learning and fear memory were evaluated by Morris water maze (MWM) and passive avoidance learning (PAL) tests, respectively. In diabetic rats, escape latency (the time is taken to reach the hidden platform in each trial day) was higher than control rats, and time spent in the target quadrant in probe trial was less than control rats. In the PAL test, step-through latency (STL) in the diabetic group was less than control rats, and the time spent in the dark compartment (TDC) was higher than control rats in the retention test^23^. All of the results are based on previous research that diabetes causes memory impairment^30,31^. We revealed that QCSPIONs even at lower concentrations than pure QC lead to obtaining better results in improved learning and memory of diabetic rats by reduction of escape latency over training trials, enhancement of the time spent in the target quadrant in the probe trial, an enhancement of STL, and reduction of TDC in the retention test^23^. In a separate study, we concluded that SPION as a targeted drug delivery system enhances the bioavailability of QC in the brain of healthy rats about ten folds higher than free QC and could be used for the treatment of neurodegenerative disorders^27^. In line with these findings, the focus of the present study was to clarify one of the underlying mechanisms of the neuroprotective effect of QCSPIONs on diabetes-induced learning and memory impairment in rats. First, it should be considered whether these SPIONs cross the BBB or not. ICP- AES method was used for the quantitative measurement of nanoparticles in the brain. The result demonstrated that SPIONs crossed the BBB and internalized in nervous system cells (Fig. 1). Cengelli et al. have proposed that the BBB damage in neurodegenerative diseases may facilitate the entrance of SPIONs to the brain endothelial cells of rats^32^. Based on this study and the relationship between diabetes mellitus and BBB dysfunction^33^, it can be concluded that diabetes by damage to the BBB facilitates the entry of nanoparticles into the brain.

Since 2007, several studies have described the role of Nrf2 in various diseases such as metabolic syndrome, obesity, retinopathy, nephropathy, and neuropathy, where its activation prevents the development of diabetes and its complications^34^. It has been demonstrated that natural compounds derived from plants and vegetables such as curcumin, resveratrol, and QC can activate Nrf2 and, thus, promote antioxidant pathways to mitigate oxidative stress and hyperglycemic damage^34^. In the present study, we observed that uncontrolled diabetes significantly reduced the expression level of Nrf2 that is in line with previous reports (Fig. 3A). In this regard, Wang et al. showed that cardiac Nrf2 expression in DM mice significantly decreased at 3 and 6 months, coincident with impaired cardiac function. The decrease in Nrf2 expression in DM mice was attenuated by resveratrol, which also increased the levels of Nrf2’s downstream antioxidative targets^35^. Moreover, reduced Nrf2 expression in the hearts of patients with chronic diabetes was reported by Tan et al.^36^. Jiang and et al. revealed Nrf2 expression decreased in diabetic nephropathy glomeruli compared with normal glomeruli and indicated the protective role of Nrf2 in STZ-induced diabetic nephropathy^37^. Here, we observed that the Nrf2 mRNA level was increased in DM rats after oral administration of QC and QCSPIONs that are consistent with other studies that have indicated the modulation of Nrf2 via QC treatment in various diseases^38–40^. Intriguingly, SPIONs administration showed a significant increase in Nrf2 expression compared to the diabetic group, which is consistent with passive avoidance learning (PAL) results. Changes in the expression of Nrf2 by SPIONs may be one of the reasons for the improvement effect of SPIONs on memory and passive avoidance learning. To evaluate the molecular mechanism of neuroprotection QCSPIONs on diabetic rats, the expression of miRNA-27a was detected in the hippocampus. Real-time PCR analysis showed that the expression level of miR-27a in diabetic rats was tripled compared to the control group, which in turn lead to downregulation Nrf2 and its target genes (Fig. 2). These results are in agreement with those of Herrera et al. (2010) in adipose tissue of Goto-Kakizaki (GK) rats, a spontaneous rat model of T2D, and Nielsen et al. (2012) in serum of T1D patients and Zhao et al. (2018) in diabetic embryopathy who found that upregulation of miR-27a in hyperglycemia^13,41,42^. Administration of QC in both pure and conjugated forms significantly decreased miR-27a expression to control level, suggesting the anti-oxidant effect of QC by miRNA regulation. Regulation miR-27a by QC treatment in several cancers such as colon, renal and colorectal cancer have been reported^24–26^. In the current study, we also investigated the mRNA expression level of three important antioxidant enzymes by real-time PCR. The results showed downregulation in the hippocampal expression level of SOD1, GPX1, and CAT in the diabetic group compared to the control group (Fig. 3B–D). Some studies have reported the reduction of antioxidant enzyme expression and their activity under hyperglycemic conditions. For example, Chatuphonprasert in 2014 revealed diabetes decreased SOD1 and CAT activities in the brain of STZ-nicotinamide-induced type 2-diabetes however antioxidant dysregulation was restored by berberine^43^. On the other hand, a growing body of evidence showing QC acts as a potent antioxidant. Mahesh conducted one of these studies in 2004 on STZ-induced diabetic rats. The results of this study showed that the daily application of QC during 45 days lead to increased activity of CAT and SOD^44^. A recent study by Iskender indicated that QC significantly increased SIRT1, SOD, and CAT activity in the kidney of STZ-induced diabetic rats^45^. In the current study, we found that QCSPIONs were more effective than pure QC in the improvement of SOD1 and CAT expression levels. This could be a reason for the higher efficacy of QCSPIONs for improving the learning and memory of diabetic rats. This observation suggests that QCSPIONs also directly and without the intermediates of the miRNA/Nrf2 pathway affect the modulation antioxidant gene expression. Unexpectedly, the level of expression of GPX1 in the diabetic group showed a very severe decrease so that both QC and QCSPIONs treatments had no effect on its mRNA expression. In the present study, TAC as an oxidative stress index increased significantly in diabetic group compared to control whereas both QC and QCSPIONs treatments showed a normalization effect on TAC status of diabetic rats (Fig. 4). TAC is a method that estimates the potential composition of different antioxidants that interact with each other. We believe that an increase in the total antioxidant level in the diabetic group could be due to an increase in potential composition of different antioxidants, which is the key for intervention to counteract ROS induced damage and diabetic neuropathy progression. In addition, this finding is in agreement with our previous report which showed both QC and QCSPIONs groups (50 mg/kg and 100 mg/kg) didn’t show any change in hepatic TAC, GSH, MDA levels, and CAT activity of intact/healthy rats^46^. Besides, we investigated the antitoxic effects of QC and QCSPIONs against H2O2-induced toxicity in PC12 cells in a separate study. These results showed that the catalase-like activity of SPIONs remained intact after conjugation^47^. From these studies, it can be concluded that nanoparticles with minimal toxicity on the cell culture and animal model could be used as a drug delivery system in neurodegenerative diseases. It is necessary to mention that nanoparticles should be considered in terms of their side effects, safety, and toxicity. The toxicity of IONPs is affected by their properties, including shape, size, surface charge, concentration and duration, functional groups, and the type of coating^48^. Finally, histological analysis by H&E staining revealed that morphological changes in the pancreas, liver, kidney, and hippocampus of diabetic rats. Treatment with QC and QCSPIONs restored the shape of beta islands, prevented glomeruli deformation, preserved the liver lobular architecture, and prevented necrosis in dentate gyri (Fig. 5). These results are consistent with new reports that QC NPs can assist in cellular regeneration^1,49^. In addition, PAS staining showed PAS-positive inclusions in Langerhans islet cells, expansive mesangial expansion, and thickening of the glomerular basement membrane and depletion hepatic glycogen stores in diabetic rats (Fig. 6). Restoration of these histological abnormalities by QC and QCSPIONs treatments confirmed antioxidant effect QC in various diseases that have oxidative stress in common. In addition, numerous human intervention studies showed that QC is a safe antioxidant compound when used in amounts up to 500 mg twice daily for 12 weeks in humans^50^. Besides the safety of QC in the human body, chronic toxicity studies in animal models showed several adverse effects such as decreased body weights, organ toxicity, genotoxicity, increased relative organ weights, existing cancer cell promotion, and drug interaction^50^. Some evidence has been provided that high or prolonged use of QC may cause an enhancement of nephrotoxic effects and kidney damage. In this regard, one study revealed orally application of QC at a concentration of 70 mg/kg for 28 weeks leads to nephrotoxicity in diabetic rats^51^. Another study revealed chronic nephropathy in male rats, which were treated with 0.1, 1, or 4% QC in feed for 2 years^52^. When QC exerts its antioxidant activity, it might be converted into potentially harmful oxidation products such as ortho-quinone that denoted as QQ which can react with protein thiols. QQ can either recycle to QC through ascorbate or convert to 6-GSQ as well as 8-GSQ during reaction with thiol GSH. When quercetin used for long-term or in high doses, QQ reacts with protein thiols and disrupts the function of enzymes containing the thiol group^53^. Therefore, a method to reduce the term or dose of QC while maintaining its therapeutic properties seems necessary.

**Figure 7:**
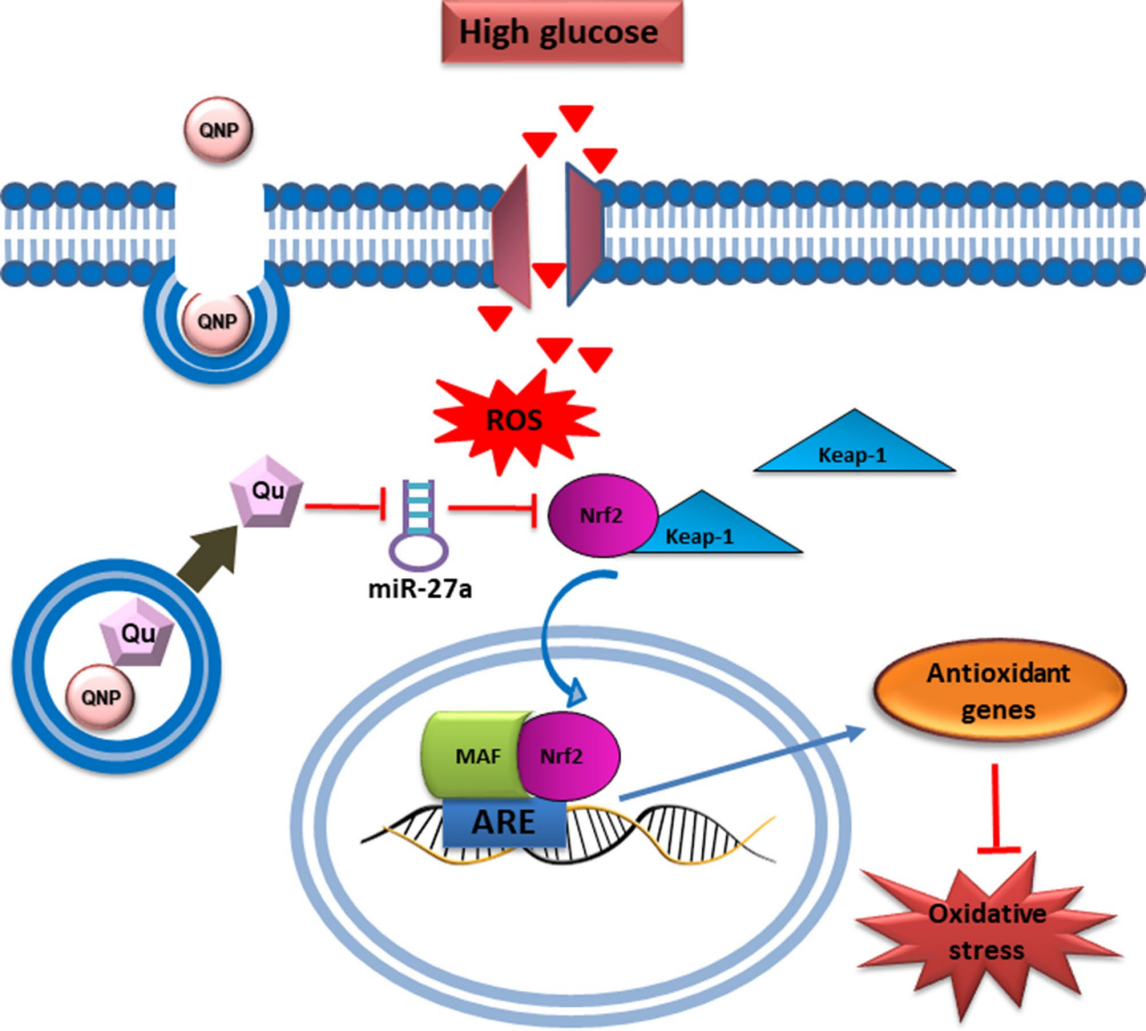
Schematic presentation of the beneficial effect of quercetin conjugated with superparamagnetic iron oxide nanoparticles on miR-27a/Nrf2-dependent antioxidant pathway in the hippocampus of diabetic rats.

In conclusion, the findings from our study demonstrate that QCSPIONs improves learning and memory in STZ-induced diabetic rats via increasing Nrf2 and antioxidant genes expression level by miR-27a regulation (Fig. 7). Despite the limitations of this study performed on the rat model of diabetes, we have revealed one of the underlying mechanisms for the protective and antioxidant roles of QCSPIONs on improving learning and memory. Additionally, this work indicates a protective role of miR-27a/Nrf2-mediated defense system in diabetic neuropathy, suggesting that dietary or therapeutic activation of Nrf2 or suppression of miR-27a could be considered as a strategy to prevent or slow down the progression of diabetic neuropathy in the future.

## Methods

### Synthesis of QCSPIONs

Synthesis of QCSPIONs referred to our previous studies^23,27^. Briefly, the chemical co-precipitation (CPT) method was used to synthesize dextran-coated Fe_3_O_4_ nanoparticles. Then QC (Sigma-Aldrich Co., St. Louis, MO, USA) was added to synthesized dextran-coated Fe_3_O_4_ NPs to make QC conjugated magnetite NPs. FTIR spectroscopy, XRD patterns, FE-SEM images were recorded.

### Experimental induction of diabetes and treatment schedule

Animal maintenance, diabetes induction, and treatment schedule were elaborated in the previous study^23^. Briefly, 40 adult male Wistar rats, weighing 200–230 g were purchased from Royan Institute (Isfahan, Iran) and maintained for 2 months in the animal holding room with the standard condition. For induction of type 1 DM, STZ was administered daily (i.p.) for 5 consecutive days at a dose of 20 mg/kg. Animals randomly divided into five groups including eight rats each: control, diabetes, and diabetes treated with SPIONs, QC, and QCSPIONs. All formulations (at the dose of 25 mg/kg) were suspended in deionized water (DI) immediately before administration and gavaged at a daily dose for a period of 35 consecutive days. 25 mg/kg was used as a standard and tested dose based on previous articles in this field which reported its beneficial effects^31,54,55^. At the end of the experiment, rats were sacrificed by ketamine-xylazine anesthesia. The hippocampus was removed from the hemispheres and ½ of that immediately frozen in liquid nitrogen and then stored at − 70 °C until use. The other half of the hippocampus and other tissues were fixed in 10% formalin and routine paraffin sections (3–4 μm) were preserved for histopathologic evaluation. The ethical respects were done in accordance with the guidelines for the use and care of research laboratory animals (USA National Institute of Health Publication No. 80-23, revised 1996) and the experimental protocols were approved by the University of Isfahan animal ethics committee.

### Iron determination in brain

To measure the concentration of iron in the brain tissue, inductively coupled plasma-atomic emission spectrometry (ICP-AES, ICPS-7500, Shimadzu, Japan) was used^56,57^. The equal amount of brain sections were prepared and treated with HNO3 (65%) for 16 h at room temperature and were filtered until a transparent solution was obtained. Using the ICP-AES technique, liquid samples were converted into aerosol form and then were disintegrated by a radio frequency field under a very high pressure, generated using Argon. The extracted ions were sent into a detector.

### RNA extraction

50–100 mg of tissue was used for each animal for RNA extraction. In order to disruption of tissue samples, squash in a sterile Petri dish was done. Total cellular RNA (including messenger RNA and microRNA) was isolated from tissue samples using the TRIzol Reagent (Invitrogen, Life Technologies, Grand Island, NY, USA) according to the manufacturer’s protocol. For complete homogenization of samples in the TRI reagent, syringe 2.5 cc with needle 18 g was used. The concentration and purity of RNA were assessed by a Nanodrop spectrophotometer (Thermo Fisher Scientific, USA). The mean absorbance ratio at 260/280 nm was 1.88 ± 0.02 and at 260/ 230 nm was 1.9 ± 0.02. Also, RNA integrity was checked by 1% denaturing agarose gel electrophoresis. To remove DNA contamination in RNA samples, all RNA samples treated with RNAase-free DNase (Thermo Fisher Scientific Inc, USA). All procedures were done under a laminar hood.

### Complementary DNA (cDNA) synthesis

cDNA from microRNAs was synthesized using BON-miR miRNA 1st strand cDNA synthesis kit (Bonyakhteh, Tehran, Iran, Cat No # BON209001). In brief, miRNAs were elongated in a polyadenylation reaction with a final volume of 20 μL at 37 °C for 30 min. Then the reaction was inactivated at 65 °C for 20 min. In the following the cDNA synthesis reaction was carried out at a final volume of 20 μL containing 10 μL polyadenylated total RNA, 1 μM Bon-RT adaptor, 1 μL RT enzyme, 2 μL dNTP mix, and 4 μL 5 × RT buffer. Reactions were incubated at 75 °C for 5 min, 25 °C for 10 min, 42 °C for 60 min, and 70 °C for 10 min.

cDNA from total RNA was performed using a PrimeScript RT reagent kit (Takara Bio, Ohtsu, Japan, Cat No # RR037A) in a final volume of 10 μL. The reaction mix contained 500 ng DNase treated total RNA, 2 μL 5 × PrimeScript buffer, 0.5 μL RT enzyme, 0.5 μL oligo dT primer, 0.5 μL of random 6mer and incubated as follows: 37 °C for 15 min and 85 °C for 5 s.

## Real-time PCR

Each synthesized cDNA was applied as a template for a distinct microRNA and mRNA quantitative real-time PCR assay using the RealQ Plus 2 × Master Mix Green (Ampliqon, Odense, Denmark). β-Actin and miR-U78 were selected as reference genes for normalization mRNA and microRNA expression levels respectively. Both of them are stable and reproducible in tissue and are appropriate reference genes for analysis of mRNA and miRNA expression. Forward and universal reverse primer of miRNA-27a were designed and synthesized by Bonyakhteh Company (Bonyakhteh, Tehran, Iran). The real-time PCR assay was carried out in a final reaction volume 13 μL containing 1 μL cDNA, 0.5 μL miRNA specific forward primer, 0.5 μL universal reverse primer, 6.5 μL QPCR master mix. qPCR reactions were performed under the following cycling conditions: 95 °C for 2 min, followed by 40 cycles of 95 °C for 5 s and 60 °C for 30 s.

All primers for mRNA samples were designed using Allele ID primer design software version 7.5 (Premier Biosoft, USA; https://premierbiosoft.com/crm/jsp/com/pbi/crm/clientside/ProductList.jsp) (Table 1). Then checked on the NCBI website (www.ncbi.nlm.nih.gov/blast) and purchased from BIONEER (City, Korea). QPCR for mRNAs assay was performed in a final reaction volume of 10 μL containing 5 μL SYBR Green I Master mix, 0.5 μM of 10 pM forward, 0.5 μM of 10 pM reverse primers and 1 μl cDNA. Real-time PCRs were done under the following cycling conditions: 15 s at 95 °C as the first denaturation step, followed by 39 cycles at 95 °C for 15 s and 57 °C for 30 s and 72 °C for 15 s. Primers and cDNA concentrations, primers efficiency and PCR conditions were optimized. It should be noted that the primers used for catalase gene expression were 1 μM. The mean threshold cycle (CT) was obtained from duplicate amplifications using a chromo4 (Bio-Rad, USA) device. The ΔΔCt method was used to compare the expression level of genes between groups.

**Table 1:** Primers for real-time PCR.

Gene	Forward primer (5′–3′)	Reverse primer	Product size (bp)
B-actin	CTCTATGCCAACACAGTG	AGGAGGAGCAATGATCTT	123
CAT	ACACTTTGACAGAGAGCGGA	TTTCACTGCAAACCCACGAG	220
SOD1	CACGAGAAACAAGATGACT	AGACTCAGACCACATAGG	129
GPX1	GGAGAATGGCAAGAATGAAGA	CGCAGGAAGGTAAAGAG	139
Nrf2	AGAGCAAGAAGCCAGATA	TCACATCACAGTAGGAAGT	103

### Total antioxidant capacity (TAC) assay

The total antioxidant capacity of the hippocampus was measured using a commercial kit following the manufacturer’s instructions (ZellBio GmbH, Germany) on the basis of the oxidation–reduction colorimetric assay at a wavelength of 490 nm. In order to the preparation tissue homogenate, 100 mg of the hippocampus of each animal was weighed, and a solution of phosphate buffer (0.1 M, pH 7.4) was added and homogenized thoroughly by a homogenizer. In order to collect supernatant, centrifugation (at 4,000 RPM, for 20-min at room temperature) was performed. The TAC index was measured using a TAC assay kit by microplate reader detection at 490 nm. TAC level was considered as the amount of antioxidant in the sample that was compared with ascorbic acid action as a standard. This method can determine TAC with 0.1 mM sensitivity (100 μmol/L).

### Histological study

To evaluate histopathological alterations, the extracted specimens including hippocampus, liver, kidney, and pancreas were instantaneously fixed in 10% formaldehyde and embedded in paraffin wax. Slides (3–4 μm) were then deparaffinized and hydrated in distilled water. According to standard protocols, sections were stained with hematoxylin–eosin (H&E), to evaluate the architecture of tissues; Periodic acid- Schiff (PAS), to access the storage of glycogen and evaluate basement membrane. Finally, histological images were taken by Leitz microscope (Nikon Eclipse E-200, Japan) with 400 times enlargement.

### Statistical analysis

The data were expressed as means ± S.E.M and analyzed by using the GraphPad Prism software package (GraphPad Software Inc., San Diego, CA, USA). Tukey one-way and two-way analysis of variance (ANOVA) were used for statistical analysis. p < 0.05 was considered statistically significant. ImageJ software was used for histologic evaluations.

## References

[1] Yang DK, Kang H-S (2018). Anti-diabetic effect of cotreatment with quercetin and resveratrol in streptozotocin-induced diabetic rats. Biomol. Ther..

[2] Asmat U, Abad K, Ismail K (2016). Diabetes mellitus and oxidative stress—a concise review. Saudi Pharm. J..

[3] Hfaiedh N, Mbarki S, Alimi H, Murat JC, Elfeki A (2013). Diabetes-induced damages in rat kidney and brain and protective effects of natural antioxidants. Food Nutr. Sci..

[4] Baglietto-Vargas D, Shi J, Yaeger DM, Ager R, LaFerla FM (2016). Diabetes and Alzheimer’s disease crosstalk. Neurosci. Biobehav. Rev..

[5] Ebrahimpour S, Zakeri M, Esmaeili A (2020). Crosstalk between obesity, DIABETES, and Alzheimer’s disease: introducing quercetin as an effective triple herbal medicine. Ageing Res. Rev..

[6] Cheng X, Ku C-H, Siow RC (2013). Regulation of the Nrf2 antioxidant pathway by microRNAs: new players in micromanaging redox homeostasis. Free Radical Biol. Med..

[7] Ma Q (2013). Role of nrf2 in oxidative stress and toxicity. Annu. Rev. Pharmacol. Toxicol..

[8] JohnsonJA J (2008). TheNrf2 ARE pathway: an indicator and modulator of oxidative stress in neurodegeneration. Ann. N. Y. Acad. Sci..

[9] Tebay LE (2015). Mechanisms of activation of the transcription factor Nrf2 by redox stressors, nutrient cues, and energy status and the pathways through which it attenuates degenerative disease. Free Radical Biol. Med..

[10] Xie Z, Wu B, Shen G, Li X, Wu Q (2018). Curcumin alleviates liver oxidative stress in type 1 diabetic rats. Mol. Med. Rep..

[11] Bryan HK, Olayanju A, Goldring CE, Park BK (2013). The Nrf2 cell defence pathway: Keap1-dependent and-independent mechanisms of regulation. Biochem. Pharmacol..

[12] Papp D (2012). The NRF2-related interactome and regulome contain multifunctional proteins and fine-tuned autoregulatory loops. FEBS Lett..

[13] Zhao Y, Dong D, Reece EA, Wang AR, Yang P (2018). Oxidative stress-induced miR-27a targets the redox gene nuclear factor erythroid 2-related factor 2 in diabetic embryopathy. Am. J. Obstet. Gynecol..

[14] Prasad KN (2017). Oxidative stress and pro-inflammatory cytokines may act as one of the signals for regulating microRNAs expression in Alzheimer’s disease. Mech. Ageing Dev..

[15] Ahn T-B, Jeon BS (2015). The role of quercetin on the survival of neuron-like PC12 cells and the expression of α-synuclein. Neural Regen. Res..

[16] Ganesan P, Ko H-M, Kim I-S, Choi D-K (2015). Recent trends in the development of nanophytobioactive compounds and delivery systems for their possible role in reducing oxidative stress in Parkinson’s disease models. Int. J. Nanomed..

[17] Nabavi SF, Russo GL, Daglia M, Nabavi SM (2015). Role of quercetin as an alternative for obesity treatment: you are what you eat!. Food Chem..

[18] de Granada-Flor, A., Sousa, C., Filipe, H., Santos, M. S. C. & de Almeida, R. F. Quercetin dual interaction at the membrane level. *Chem. Commun. *(2019). 10.1039/c8cc09656b 30664132

[19] Boots AW, Haenen GR, Bast A (2008). Health effects of quercetin: from antioxidant to nutraceutical. Eur. J. Pharmacol..

[20] Costa LG, Garrick JM, Roquè PJ, Pellacani C (2016). Mechanisms of neuroprotection by quercetin: counteracting oxidative stress and more. Oxid. Med. Cell. Long..

[21] Wang D, Sun-Waterhouse D, Li F, Xin L, Li D (2018). MicroRNAs as molecular targets of quercetin and its derivatives underlying their biological effects: A preclinical strategy. Crit. Rev. Food Sci. Nutr..

[22] Kim MK, Park K-S, Yeo W-S, Choo H, Chong Y (2009). In vitro solubility, stability and permeability of novel quercetin–amino acid conjugates. Bioorg. Med. Chem..

[23] Ebrahimpour S, Esmaeili A, Beheshti S (2018). Effect of quercetin-conjugated superparamagnetic iron oxide nanoparticles on diabetes-induced learning and memory impairment in rats. Int. J. Nanomed..

[24] Del Follo-Martinez A, Banerjee N, Li X, Safe S, Mertens-Talcott S (2013). Resveratrol and quercetin in combination have anticancer activity in colon cancer cells and repress oncogenic microRNA-27a. Nutr. Cancer.

[25] Li W (2014). Combination of quercetin and hyperoside has anticancer effects on renal cancer cells through inhibition of oncogenic microRNA-27a. Oncol. Rep..

[26] Toden S (2015). Novel evidence for curcumin and boswellic acid–induced chemoprevention through regulation of miR-34a and miR-27a in colorectal cancer. Cancer Prev. Res..

[27] Najafabadi RE, Kazemipour N, Esmaeili A, Beheshti S, Nazifi S (2018). Using superparamagnetic iron oxide nanoparticles to enhance bioavailability of quercetin in the intact rat brain. BMC Pharmacol. Toxicol..

[28] Zilliox LA, Chadrasekaran K, Kwan JY, Russell JW (2016). Diabetes and cognitive impairment. Curr. Diabetes Rep..

[29] Testa R, Bonfigli A, Genovese S, De Nigris V, Ceriello A (2016). The possible role of flavonoids in the prevention of diabetic complications. Nutrients.

[30] Bhutada P (2010). Ameliorative effect of quercetin on memory dysfunction in streptozotocin-induced diabetic rats. Neurobiol. Learn. Mem..

[31] Maciel RM (2016). Neuroprotective effects of quercetin on memory and anxiogenic-like behavior in diabetic rats: role of ectonucleotidases and acetylcholinesterase activities. Biomed. Pharmacother..

[32] Cengelli F (2006). Interaction of functionalized superparamagnetic iron oxide nanoparticles with brain structures. J. Pharmacol. Exp. Ther..

[33] Prasad S, Sajja RK, Naik P, Cucullo L (2014). Diabetes mellitus and blood-brain barrier dysfunction: an overview. J. Pharm..

[34] Jiménez-Osorio AS, Gonzalez-Reyes S, Pedraza-Chaverri J (2015). Natural Nrf2 activators in diabetes. Clin. Chim. Acta.

[35] Wang G, Song X, Zhao L, Li Z, Liu B (2018). Resveratrol prevents diabetic cardiomyopathy by increasing Nrf2 expression and transcriptional activity. BioMed Res. Int..

[36] Tan Y (2011). Diabetic downregulation of Nrf2 activity via ERK contributes to oxidative stress–induced insulin resistance in cardiac cells in vitro and in vivo. Diabetes.

[37] Jiang T (2010). The protective role of Nrf2 in streptozotocin-induced diabetic nephropathy. Diabetes.

[38] Mostafavi-Pour Z, Ramezani F, Keshavarzi F, Samadi N (2017). The role of quercetin and vitamin C in Nrf2-dependent oxidative stress production in breast cancer cells. Oncol. Lett..

[39] Granado-Serrano AB, Martín MA, Bravo L, Goya L, Ramos S (2012). Quercetin modulates Nrf2 and glutathione-related defenses in HepG2 cells: involvement of p38. Chem. Biol. Interact..

[40] Marina R, González P, Ferreras MC, Costilla S, Barrio JP (2015). Hepatic Nrf2 expression is altered by quercetin supplementation in X-irradiated rats. Mol. Med. Rep..

[41] Herrera B (2010). Global microRNA expression profiles in insulin target tissues in a spontaneous rat model of type 2 diabetes. Diabetologia.

[42] Nielsen LB (2012). Circulating levels of microRNA from children with newly diagnosed type 1 diabetes and healthy controls: evidence that miR-25 associates to residual beta-cell function and glycaemic control during disease progression. Exp. Diabetes Res..

[43] Chatuphonprasert W, Lao-Ong T, Jarukamjorn K (2014). Improvement of superoxide dismutase and catalase in streptozotocin–nicotinamide-induced type 2-diabetes in mice by berberine and glibenclamide. Pharm. Biol..

[44] Mahesh T, Menon VP (2004). Quercetin allievates oxidative stress in streptozotocin-induced diabetic rats. Phytother. Res..

[45] Iskender H (2017). The effect of hesperidin and quercetin on oxidative stress, NF-κB and SIRT1 levels in a STZ-induced experimental diabetes model. Biomed. Pharmacother..

[46] Kazemipour N (2018). Hepatotoxicity and nephrotoxicity of quercetin, iron oxide nanoparticles, and quercetin conjugated with nanoparticles in rats. Comp. Clin. Pathol..

[47] Yarjanli Z, Ghaedi K, Esmaeili A, Zarrabi A, Rahgozar S (2019). The antitoxic effects of quercetin and quercetin-conjugated iron oxide nanoparticles (QNPs) against H2O2-induced toxicity in PC12 cells. Int. J. Nanomed..

[48] Yarjanli Z, Ghaedi K, Esmaeili A, Rahgozar S, Zarrabi A (2017). Iron oxide nanoparticles may damage to the neural tissue through iron accumulation, oxidative stress, and protein aggregation. BMC Neurosci..

[49] Saedi E, Gheini MR, Faiz F, Arami MA (2016). Diabetes mellitus and cognitive impairments. World J. Diabetes.

[50] Andres S (2018). Safety aspects of the use of quercetin as a dietary supplement. Mol. Nutr. Food Res..

[51] Hsieh C-L (2010). Quercetin and ferulic acid aggravate renal carcinoma in long-term diabetic victims. J. Agric. Food Chem..

[52] Dunnick JK, Halley JR (1992). Toxicity and carcinogenicity studies of quercetin, a natural component of foods. Toxicol. Sci..

[53] Boots AW, Kubben N, Haenen GR, Bast A (2003). Oxidized quercetin reacts with thiols rather than with ascorbate: implication for quercetin supplementation. Biochem. Biophys. Res. Commun..

[54] Denny Joseph K (2015). Combined oral supplementation of fish oil and quercetin enhances neuroprotection in a chronic rotenone rat model: relevance to Parkinson's Disease. Neurochem. Res..

[55] Pattanashetti LA, Taranalli AD, Parvatrao V, Malabade RH, Kumar D (2017). Evaluation of neuroprotective effect of quercetin with donepezil in scopolamine-induced amnesia in rats. Indian J. Pharmacol..

[56] Ebrahimpour, S., Shahidi, S. B., Aabbasi, M., Tavakoli, Z. & Esmaeili, A. Quercetin-conjugated superparamagnetic iron oxide nanoparticles enhances Nrf2 expression through miR-27a intervention to prevent memory dysfunction in diabetic rats. (2020). 10.1038/s41598-020-71971-2 PMC7524758 32994439

[57] Katebi S, Esmaeili A, Ghaedi K, Zarrabi A (2019). Superparamagnetic iron oxide nanoparticles combined with NGF and quercetin promote neuronal branching morphogenesis of PC12 cells. Int. J. Nanomed..

